# Volume matters in the systemic treatment of metastatic pancreatic cancer: a population-based study in the Netherlands

**DOI:** 10.1007/s00432-016-2140-5

**Published:** 2016-03-19

**Authors:** N. Haj Mohammad, N. Bernards, M. G. H. Besselink, O. R. Busch, J. W. Wilmink, G. J. M. Creemers, I. H. J. T. De Hingh, V. E. P. P. Lemmens, H. W. M. van Laarhoven

**Affiliations:** 1 Department of Medical Oncology, Academic Medical Center, Meibergdreef 9, 1100 DD Amsterdam, The Netherlands; 2 Department of Medical Oncology, Catharina Hospital, Michelangelolaan 2, 5623 EJ Eindhoven, The Netherlands; 3 Department of Research, Comprehensive Cancer Organisation The Netherlands/Netherlands Cancer Registry, Godebaldkwartier 419, 3511 DT Utrecht, The Netherlands; 4 Department of Surgery, Academic Medical Center, Meibergdreef 9, 1100 DD Amsterdam, The Netherlands; 5 Department of Surgical Oncology, Catharina Hospital, Michelangelolaan 2, 5623 EJ Eindhoven, The Netherlands; 6 Department of Public Health, Erasmus Medical Center, Wytemaweg 80, 3015 CN Rotterdam, The Netherlands

**Keywords:** Pancreatic cancer, High-volume hospital, Palliative chemotherapy, Survival, Population based

## Abstract

**Purpose:**

In pancreatic surgery, a relation between surgical volume and postoperative mortality and overall survival (OS) has been recognized, with high-volume centers reporting significantly better survival rates. We aimed to explore the influence of hospital volume on administration of palliative chemotherapy and OS in the Netherlands.

**Methods:**

Patients diagnosed between 2007 and 2011 with metastatic pancreatic cancer were identified in the Netherlands Cancer Registry. Three types of high-volume centers were defined: high-volume (1) incidence center, based on the number of patients diagnosed with metastatic pancreatic cancer, (2) treatment center based on number of patients with metastatic pancreatic cancer who started treatment with palliative chemotherapy and (3) surgical center based on the number of resections with curative intent for pancreatic cancer. Independent predictors of administration of palliative chemotherapy were evaluated by means of logistic regression analysis. The multivariable Cox proportional hazard model was used to assess the impact of being diagnosed or treated in high-volume centers on survival.

**Results:**

A total of 5385 patients presented with metastatic pancreatic cancer of which 24 % received palliative chemotherapy. Being treated with chemotherapy in a high-volume chemotherapy treatment center was associated with improved survival (HR 0.76, 95 % CI 0.67–0.87). Also, in all patients with metastatic pancreatic cancer, being diagnosed in a high-volume surgical center was associated with improved survival (HR 0.74, 95 % CI 0.66–0.83).

**Conclusions:**

Hospital volume of palliative chemotherapy for metastatic pancreatic cancer was associated with improved survival, demonstrating that a volume–outcome relationship, as described for pancreatic surgery, may also exist for pancreatic medical oncology.

**Electronic supplementary material:**

The online version of this article (doi:10.1007/s00432-016-2140-5) contains supplementary material, which is available to authorized users.

## Introduction

The incidence of pancreatic cancer is rising in developed countries. In 2012 alone, 104,481 individuals died from pancreatic cancer making it the fifth leading cause of cancer death with the worst overall survival (European Cancer Observatory 2012). By 2030, pancreatic cancer is projected to become the second leading cause of cancer-related death (Rahib et al. 2014). The only potentially curative treatment for pancreatic cancer is surgical resection. However, only about 20 % of the patients present with resectable disease. Patients not fit enough to undergo surgery or patients with irresectable or metastatic tumors have a poor prognosis with a median survival of about three months when untreated (Royal 2011). In 1997, gemcitabine monotherapy became the first-line standard treatment. In numerous trials over the years, many different drug regimens have been tested. None of these trials demonstrated a statistically significant survival benefit, except for gemcitabine plus erlotinib, which was associated with a very modest, clinically irrelevant increase in overall survival of 2 weeks (Moore et al. 2007). Only recently two trials changed the landscape of palliative pancreatic cancer treatment, showing a substantial improvement in survival after combination chemotherapy (Conroy et al. 2011; Von Hoff et al. 2013; Goldstein et al. 2015).

The scarce availability of treatment options may have led to reserved prescription as well as heterogeneity in prescription of palliative chemotherapy between hospitals. Indeed, a recently published study conducted in the Netherlands showed a large inter-hospital variation in the administration of chemotherapy (5–34 %) to patients with metastatic pancreatic carcinoma (Bernards et al. 2015). The most frequently mentioned reasons for not offering palliative chemotherapy were the age and socioeconomic status of the patient (Krzyzanowska et al. 2003; Kao et al. 2009). Furthermore, preferences and experience of medical oncologists may play an important role in the choice to start palliative therapy (Schildmann et al. 2013). The willingness to apply a treatment which may have limited benefit and potential side effects has been related to the annual number of patients qualifying for such treatment by a physician (Krammer and Heinzerling 2014).

In pancreatic surgery, a direct relation between the number of operated patients in a hospital and postoperative outcome has been established, with postoperative mortality after pancreaticoduodenectomy being significantly lower in high-volume compared to low-volume hospitals (Birkmeyer et al. 1999; Gooiker et al. 2011, 2014; Tol et al. 2012). Interestingly, in this relation between volume and outcome the overall hospital volume has been shown to be even more relevant than the volume of patients treated per individual surgeon (Gouma et al. 2000), indicating the importance of experienced non-surgical support for this specific group of patients. Similarly, it may be hypothesized that for treatment of patients with metastatic cancer, the experience of medical oncologists, as defined by the number of annually treated patients, as well as the combined experience of the whole multidisciplinary team providing pancreatic cancer care may be a relevant factor determining patient outcome.

Recently, in 245 patients with resectable pancreatic cancer a superior survival was found for patients being treated with adjuvant chemotherapy in a high-volume hospital compared to patients treated in a low-volume hospital (Mandelson and Picozzi 2016). These data were presented at ASCO Gastrointestinal Cancers Symposium 2016 and underline that further elaboration is necessary on differences in patterns of care and their impact on survival.

To our knowledge, no information is available on the relation between volume and survival of patients with metastatic pancreatic cancer. Therefore, we conducted a nationwide population-based study in patients with metastatic pancreatic cancer to assess whether volume is a predictor for (1) the percentage of patients receiving palliative chemotherapy and (2) overall survival.

## Methods

### Data collection

Data were obtained from the Netherlands Cancer Registry (NCR). This is a population-based prospective database that collects information on all patients newly diagnosed with a malignancy in the Netherlands. The registry area includes about 16.7 million inhabitants and encompasses 91 hospitals, of which eight academic. The NCR is notified by the national automated pathological archive, if the newly diagnosed cancer is microscopically verified. In the absence of verification, notification occurs by additional sources, such as the national registry of hospital discharge, multidisciplinary team reports and diagnosis therapy combinations (specific codes for reimbursement purposes). Within 6–9 months after notification, trained registration clerks, operating on behalf of the NCR, extract patient and tumor characteristics from medical records. Data are coded according to a national manual, and cancer topography and morphology are classified according to the International Classification of Disease for Oncology (ICD-O) second or third edition.

Our inclusion was limited to patients diagnosed with an adenocarcinoma, a not otherwise specified carcinoma (ICD-O morphology codes 8010, 8012, 8020, 8140,8141, 8260, 8310, 8440, 8470, 8480, 8481, 8490, 8500, 8560) or a non-microscopic verified neoplasm of the pancreas between January 2007 and December 2011. Other morphology codes were excluded or did not occur during the study period. Patients diagnosed at autopsy were excluded. Carcinomas were classified according to the Tumor Lymph Node Metastases classification and staged according to the recommendations of the International Union Against Cancer (UICC) TNM classification in the respective period. For staging of neoplasm without microscopic verification, the clinical extent of disease (cEOD) was used.

To assess the influence of hospital volume on outcome, we defined high-volume centers based on the upper quartile (Q3/75th percentile). Each volume threshold dichotomized the data and created two categories for comparison: hospitals with volume greater or equal to the cutoff value and hospitals with volume less than the cutoff value. We defined three different types of high-volume centers.

1. High-volume incidence center: a hospital volume that refers to the number of patients diagnosed with metastatic pancreatic cancer. This may be regarded the most straightforward hospital volume. However, as pancreatic cancer treatment may be an important determinant of outcome of pancreatic cancer patients and a high-volume incidence center does not necessarily treat a high volume of patients, we also identified high-volume treatment center; ≥101 patients diagnosed in 5 years (range 14–183);
2. High-volume treatment center: a hospital volume that refers to the number of metastatic pancreatic cancer patients treated with chemotherapy. This may be regarded as a proxy for the experience of a hospital to deliver care to this patient population that may develop specific complications in this disease stage; ≥28 patients treated in 5 years (range 1–116);
3. High-volume surgical center: a hospital volume which refers to the number of surgical procedures in pancreatic cancer, which may be regarded as a proxy for the presence of a well-developed infrastructure to deliver complex care to pancreatic cancer patients; ≥68 resections with curative intent treated in 5 years (range 1–123).

Vital status of all patients on January 1, 2014, was assessed through linkage with civil municipal registries and the Central Bureau for Genealogy, which collects data on all citizens who die.

### Statistical analysis

We performed all statistical analyses using SAS statistical software (version 9.4, SAS institute, Cary, NC, USA). Two sided *P* values of <0.05 were considered statistically significant.

The proportion of patients treated with chemotherapy was described for different subgroups and high-volume centers. Differences between subgroups were tested by means of a *χ* ^2^ test, and trends overtime were analyzed by means of a Cochran–Armitage trend test. Independent influences on prescription of palliative chemotherapy were evaluated by means of a logistic regression analysis. The different types of high-volume centers were added separately to the model.

Survival time was defined as the time from diagnosis to death or January 1, 2014, for patients who were still alive. The crude survival was calculated with the life test, and differences between survival curves were evaluated by means of a log-rank test. The independent prognostic effect of being diagnosed or treated in a high-volume center was estimated using Cox regression analyses.

The hazard rates for death were adjusted for gender, age, extent of disease and period of diagnosis. The influence of being diagnosed in a high-volume treatment center was investigated in patients treated with chemotherapy only; untreated patients were excluded from this analysis. In the other models chemotherapy was added separately to investigate the effect of treatment on the hazard ratio of death.

## Results

**Fig. 1:**
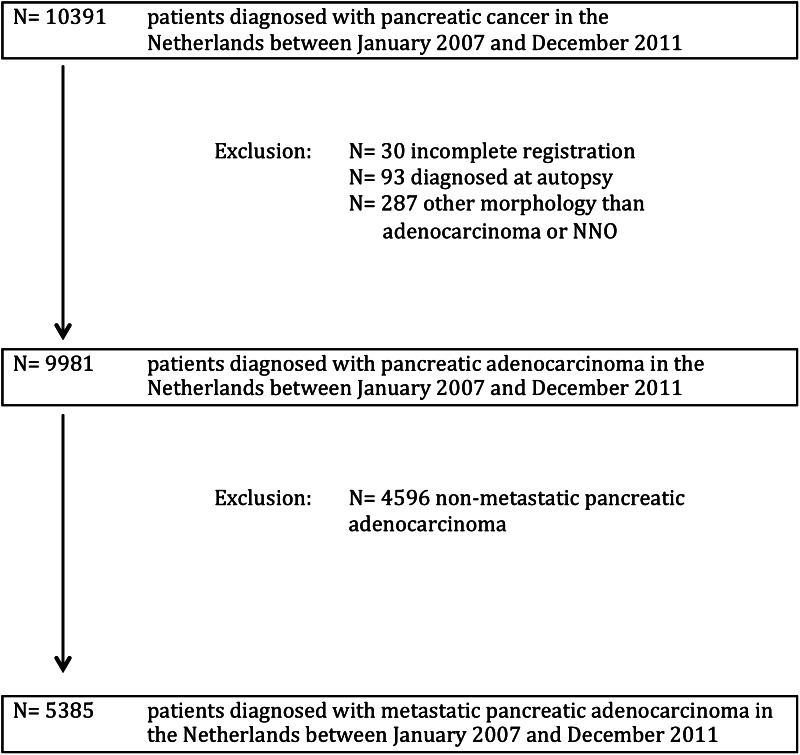
Flow diagram of included patients. *NNO* not otherwise specified

Between January 1, 2007, and December 31, 2011, 9981 patients were diagnosed with pancreatic cancer in the Netherlands, of whom 5385 (54 %) patients presented with metastatic disease. The patient selection is shown in Fig. 1. Fifty-two percent of the patients with metastatic disease were male, the median age at time of diagnosis was 69 years (range 21–100), and in 68 % of the cases the diagnosis was microscopically confirmed. The general characteristics of patients treated in high-volume centers are shown in Table 1.

**Table 1:** General characteristics of patients diagnosed with a neoplasm of the pancreas between 2007 and 2011 in the Netherlands, stratified according to high-volume center (*N* = 5385)

	Total *N* (%)	High-volume incidence center *N* (%)	High-volume treatment center *N* (%)	High-volume surgical center *N* (%)
Sex				
Male	2796 (52)	825 (54)	455 (55)	215 (59)
Female	2589 (48)	707 (46)	380 (46)	148 (41)
Age (years)				
<50	239 (4)	68 (4)	47 (6)	29 (8)
50–59	817 (15)	228 (15)	140 (17)	83 (23)
60–69	1671 (31)	505 (33)	302 (36)	131 (36)
70–79	1731 (32)	471 (31)	250 (30)	97 (27)
≥80	927 (17)	260 (17)	96 (12)	23 (6)
Histologic subtype				
Adenocarcinoma	3640 (68)	1082 (71)	659 (79)	312 (86)
Non-microscopic verified	1745 (32)	450 (29)	176 (21)	51 (14)
Location of metastases				
Liver	2770 (51)	775 (51)	407 (49)	168 (46)
Peritoneum	425 (8)	110 (7)	54 (7)	36 (10)
Lung	244 (5)	66 (4)	36 (4)	14 (4)
Extra regional lymphnodes	179 (3)	61 (4)	38 (5)	27 (7)
Other	100 (2)	25 (2)	18 (2)	7 (2)
2 organs	1190 (22)	340 (22)	199 (24)	84 (23)
3 or more organs	431 (8)	131 (9)	73 (9)	23 (6)
Chemotherapy				
Yes	1274 (24)	400 (26)	329 (39)	100 (28)
No	4111 (76)	1132 (74)	506 (61)	263 (73)
Total	5385 (100)	1532 (28)	835 (16)	363 (7)

We defined high-volume centers, based on three different volume thresholds. In, total, 17 hospitals were classified as a high-volume center. Thirteen hospitals were classified as high-volume incidence center, seven hospitals as high-volume treatment center, and four hospitals as high-volume surgical center. Only one hospital qualified for all three high-volume definitions. Another four high-volume incidence centers were also high-volume treatment centers.

Twenty-four percent (*N* = 1274) of the patients with metastatic pancreatic cancer received palliative chemotherapy. Table 2 shows the crude proportions of patients treated with chemotherapy in the different high-volume centers. The odds of receiving palliative chemotherapy were higher in high-volume treatment centers. Palliative chemotherapy was not administered more often in high-volume incidence centers or high-volume surgical centers. Other predictive factors for prescription of chemotherapy were younger age at time of diagnosis, the presence of microscopic verification [OR 3.13 (2.63–3.85)], two sites of metastases [OR 0.73 (0.57–0.94)] and a more recent year of diagnosis [OR for 2011, 2007 reference 1.55 (1.24–1.94)].

**Table 2:** Crude percentages and adjusted odds for receiving chemotherapy among patients diagnosed with metastatic pancreatic cancer between 2007 and 2011 in the Netherlands (*N* = 5385)

	Crude percentage %	Multivariate odds ratio (95 % CI)
Diagnosed in high-volume incidence center^a^		
Yes	26	1.14 (0.98–1.32)
No	23	1.00 (reference)
Treated in high-volume treatment center^a^		
Yes	39	2.20 (1.85–2.61)
No	21	1.00 (reference)
Diagnosed in high-volume surgical center^a^		
Yes	28	0.82 (0.64–1.07)
No	23	1.00 (reference)

^a^Different types of high-volume centers were added separately to the model adjusted for tumor and patient characteristics

We found that patients diagnosed in the hospital that qualified for all three high-volume definitions had PA verification more often compared to patients that were diagnosed in a hospital that was only one type of high-volume center (supplementary table 1). However, patients in that specific hospital were not treated with palliative chemotherapy more frequently compared to other high-volume hospitals.

The median overall survival of patients with metastatic pancreatic cancer was 9.6 weeks (1-year survival rate 6 %). Table 3 shows the results of a multivariable proportional hazard regression analysis modeling the risk of death for patients with metastatic pancreatic cancer. Factors that were associated with poor survival were older age (≥80 years), the absence of microscopic verification and metastases in multiple organs. Metastases limited to the lungs or limited to extra regional lymph nodes, treatment with palliative chemotherapy and treatment in a high-volume surgical center were identified as beneficial prognostic factors. By excluding treatment with chemotherapy from the model (result not shown), the beneficial effect of younger age was statistically significant.

**Table 3:** Crude median overall survival, crude 1-year survival and adjusted hazard ratios for patients diagnosed with metastatic pancreatic cancer between 2007 and 2011 in the Netherlands (*N* = 5385)

	Crude median survival (weeks)	Crude 1-year survival (%)	Multivariable HR (95 % CI)
Diagnosed in high-volume incidence center^a^			
Yes	9.9	6.7	0.86 (0.94–1.06)
No	9.4	5.5	1.00 (reference)
Treated in high-volume treatment center^a,b^			
Yes	28.4	21.3	0.76 (0.67–0.87)
No	22.9	11.6	1.00 (reference)
Diagnosed in high-volume surgical center^a^			
Yes	14.7	11.9	0.74 (0.66–0.83)
No	9.3	5.4	1.00 (reference)

^a^Different types of high-volume centers were added separately to the model adjusted for all the above listed variables

^b^Only patients treated with palliative chemotherapy were included in the analysis (*N* = 1274)

The median overall survival in patients treated with palliative chemotherapy was 24 weeks (1-year survival rate (14 %). Multivariable hazard regression analysis in patients treated with palliative chemotherapy revealed that being treated in a high-volume treatment center was associated with improved survival (HR 0.76, 95 % CI 0.67–0.87) (Table 3).

## Discussion

To the best of our knowledge, this is the first study showing a positive correlation between hospital volume and overall survival in patients with metastatic pancreatic cancer. The population-level findings demonstrate that being diagnosed in a high-volume surgical center and being treated with palliative chemotherapy in a high-volume treatment center are associated with improved survival.

The presence of microscopic verification of (metastatic) pancreatic cancer, as well as younger age, is well-known and established predictors for starting palliative chemotherapy (Krzyzanowska et al. 2003). However, hospital volume (high-volume treatment center) as a predictor for starting palliative chemotherapy has never been reported before. Patients that were treated with palliative chemotherapy in a center that had a high treatment volume of pancreatic cancer patients exhibited a better survival than patients that were treated with palliative chemotherapy in other hospitals.

The association between high-volume treatment center and prolonged survival may be explained by the experience of high-volume treatment centers with this specific patient population and specific chemotherapy treatment. Already in 1979, Luft et al. (1979) described for pancreatic surgery an inverse relation between surgical volume and mortality. Since centralization of pancreatic surgery, a relation between surgical volume, postoperative mortality and overall survival has been demonstrated, with high-volume surgical centers reporting significantly better survival rates (Birkmeyer et al. 2002; van Oost et al. 2006; Gooiker et al. 2011).

In metastatic disease, the medical oncologist has to weigh patient’s prognosis, treatment toxicity and the possible positive impact on quality or quantity of life and together with the patient decide to start palliative chemotherapy or to provide supportive care only. Given the often poor clinical condition of pancreatic cancer patient which may deteriorate rapidly, this decision making process is not trivial. Moreover, when starting palliative chemotherapy, toxicity has to be managed adequately, including appropriate reductions in chemotherapy dose, without stopping treatment either too early or too late. Hence, experience in treating this patient population, acquired by treating a relatively high number of patients, may be of paramount importance for the outcome of these patients. Furthermore, we hypothesize that not only the expertise of an individual medical oncologist, but also the complete infrastructure of the hospital may relate to patient outcome (Wolfson et al. 2015).

Specific tumor and treatment-related complications such as pain management, nutritional care and biliary drainage request comprehensive care. For example, cholangitis due to compression of the bile ducts and duodenal obstruction by a cancer in the pancreatic head is common in patients with metastatic cancer. Decompression has shown to improve quality of life and may improve survival (Ballinger et al. 1994; Chu and Adler 2010). A yearly volume of ≥50 ERCPs per endoscopist was significantly associated with a lower risk of procedural failure (Ekkelenkamp et al. 2015). Our population-level finding that for all patients with metastatic pancreatic cancer, being diagnosed in a high-volume surgical center and high-volume treatment center was associated with improved survival, suggests that an experienced multidisciplinary team may contribute to optimal treatment of metastatic pancreatic cancer.

The prognosis of patients with pancreatic cancer remains poor with a median survival of 9.6 weeks. It is difficult to compare this result with the outcome of randomized controlled trials, which show higher survival rates due to inclusion of relatively young patients with a good performance status. However, the median duration in survival that we observed was comparable to another population-based study (Baxter et al. 2007) but lower than other population-based studies, albeit marginally (Cress et al. 2006; Worni et al. 2013). This may be explained by the inclusion of pathologically unverified carcinomas in our study. A significant portion of patients did not have their diagnosis confirmed by pathological examination; possibly because these patients were considered unfit for palliative chemotherapy and pathological confirmation would not have had therapeutic implications. The overall survival of these patients was 7 weeks, and therefore, the likelihood that these patients indeed suffered from pancreatic cancer is high. In our series, 24 % of the patients with metastatic pancreatic cancer were treated with palliative chemotherapy. The reported percentage in previous population-based studies in metastatic pancreatic is highly variable. Moreover, the presented specific subsets of patients treated with palliative chemotherapy were inconsistent. David et al. (2009) described 30 % of the patients receiving palliative chemotherapy after palliative surgery. In non-resected patients, the use was only 17 %. Sharp et al. (2009) reported 20 %. One Australian cohort observed that 43 % of the patients received palliative chemotherapy; however, this was a smaller study with 1863 patients and selection bias may have occurred (Burmeister et al. 2015). A second Australian study by Jefford et al. (2010) reported 54 % chemotherapy use, but analyzed both resectable and irresectable patients. A recent study by Oberstein et al. (2013) seems to report a considerably higher percentage of patients treated with palliative chemotherapy (54 %). However, patients who died within 30 days (22 %) were excluded from the analysis. This implies that 42 % was treated with palliative chemotherapy. Median survival in patients treated with palliative chemotherapy was 24 weeks. This corresponds well with previously published data from the south of the Netherlands (Bernards et al. 2015). Similar to previous studies, we found that younger age and limited metastases were related to better survival (Cress et al. 2006; Tas et al. 2013).

It should be noted that this analysis was conducted in the era before FOLFIRINOX and nab-paclitaxel with gemcitabine. With the introduction of the two new combination regimens for metastatic pancreatic cancer, FOLFIRINOX and nab-paclitaxel with gemcitabine, experience of the medical oncologist may become even more important. The combination therapies have different efficacies, different side effects and different routes of administration (Conroy et al. 2011; Von Hoff et al. 2013; Goldstein et al. 2015). As there are no direct data comparing the two combination treatment regimens, experience with all known palliative treatment options is of utmost importance to select the appropriate treatment for each individual patient. Because of the higher toxicity of the combination therapy, few patients will be considered fit for the combination regimen and, as a consequence, the experience per center with a specific regimen may further decrease. Thus, concentration of medical oncological care for patients with metastatic pancreatic cancer may become even more important.

It should be acknowledged that even with very careful analysis of population-based data it cannot be excluded that part of our results are explained by selection bias. Patients treated in high-volume surgical centers may be a selection of fit patients with limited metastatic tumor load. Part of these patients may initially have been considered resectable while during explorative laparotomy patients were shown to have irresectable disease, for instance due to small peritoneal metastases. Also, it may be argued that younger, fitter patients select high-volume hospitals. To minimize the confounding effect of palliative chemotherapy itself only patients were analyzed that were treated with palliative chemotherapy. Yet, other confounders related to usage of palliative chemotherapy cannot be ruled out. However, after adjustment for patient and tumor characteristics, we showed that survival was better in high-volume treatment centers. Unfortunately, we did not have information on performance status available in our dataset (Boeck et al. 2007). This lack of information on performance status is a significant limitation of our study. Furthermore, the hospital volumes that we defined in our study need validation, and from these data no definite conclusions can be drawn on whether a specific type of high-volume center should be the norm for best clinical practice.

In conclusion, in this nationwide database, hospital volume of palliative chemotherapy was associated with improved survival demonstrating that a volume–outcome relationship, as described for pancreatic surgery, may also exist for pancreatic medical oncology.

## Electronic supplementary material

Below is the link to the electronic supplementary material.

Supplementary material 1 (DOCX 59 kb)

Supplementary material 2 (DOCX 48 kb)

Supplementary material 3 (DOCX 49 kb)

Supplementary material 4 (DOCX 49 kb)
