# Supplementary material for: Volume matters in the systemic treatment of metastatic pancreatic cancer: a population-based study in the Netherlands
Source: J Cancer Res Clin Oncol. 2016 Mar 19;142(6):1353–60. doi: 10.1007/s00432-016-2140-5 (PMC4869755; doi:10.1007/s00432-016-2140-5)
Supplement: Supplementary file 1 — Supplementary material 1 (DOCX 59 kb) [file 432_2016_2140_MOESM1_ESM.docx]

|  | High-volume  1x  N (%) | High-volume  2x  N (%) | High-volume  3x  N (%) |
| --- | --- | --- | --- |
| Sex  Male  Female | 657 (53)  576 (47) | 266 (52)  241 (48) | 102 (63)  59 (37) |
| Age (yrs)  <50  50-59  60-69  70-79  ≥80 | 63 (5)  199 (16)  372 (30)  375 (30)  224 (18) | 21 (4)  72 (14)  190 (37)  160 (32)  64 (13) | 13 (8)  36 (22)  62 (38)  41 (25)  9 (6) |
| Histologic subtype  Adenocarcinoma  Non-microscopic verified | 862 (70)  371 (30) | 378 (75)  129 (25) | 145 (90)  16 (10) |
| Location of metastases  Liver  Peritoneum  Lung  Extra regional lymphnodes  Other  2 organs  3 or more organs | 647 (52)  104 (8)  45 (4)  51 (4)  23 (2)  261 (21)  86 (7) | 233 (46)  30 (6)  25 (5)  18 (4)  12 (2)  127 (25)  54 (11) | 79 (49)  12 (7)  7 (4)  13 (8)  1 (1)  36 (22)  11 (7) |
| Chemotherapy  Yes  No | 282 (23)  951 (77) | 191 (38)  316 (62) | 55 (34)  106 (66) |
| Total | 1233 | 507 | 161 |

Supplementary table 1a Patient characteristics specified per high volume-hospital: one type high-volume hospital, two types high-volume hospital and three types high-volume hospital
