# Supplementary material for: Volume matters in the systemic treatment of metastatic pancreatic cancer: a population-based study in the Netherlands
Source: J Cancer Res Clin Oncol. 2016 Mar 19;142(6):1353–60. doi: 10.1007/s00432-016-2140-5 (PMC4869755; doi:10.1007/s00432-016-2140-5)
Supplement: Supplementary file 2 — Supplementary material 2 (DOCX 48 kb) [file 432_2016_2140_MOESM2_ESM.docx]

| Variable | Odds ratio | 95% CI |
| --- | --- | --- |
| Sex  Male  Female | reference  1.029 | 0.847-1.249 |
| Age (yrs)  <50  50-59  60-69  70-79  ≥80 | 1.303  1.275  reference  1.220  1.830 | 0.830-2.045  0.958-1.607  0.961-1.548  1.315-2.548 * |
| Histologic subtype  Adenocarcinoma  Non-microscopic verified | Reference  1.374 | 1.078-1.753* |
| Location of metastases  Liver  Peritoneum  Lung  Extra regional lymphnodes  Other  2 organs  3 or more organs | reference  1.307  0.636  0.852  0.899  0.789  0.676 | 0.886-1.927  0.394-1.027  0.532-1.367  0.447-1.809  0.621-1.003  0.475-0.962* |

Supplementary table 1b Multivariate binary logistic regression Patient characteristics in a once high-volume hospital (n=1233) vs more than one high-volume hospital (n=668)

* P<0.05

*Abbreviations*

*95% CI= 95% confidence interval*
