# Supplementary material for: Volume matters in the systemic treatment of metastatic pancreatic cancer: a population-based study in the Netherlands
Source: J Cancer Res Clin Oncol. 2016 Mar 19;142(6):1353–60. doi: 10.1007/s00432-016-2140-5 (PMC4869755; doi:10.1007/s00432-016-2140-5)
Supplement: Supplementary file 3 — Supplementary material 3 (DOCX 49 kb) [file 432_2016_2140_MOESM3_ESM.docx]

| Variable | Odds ratio | 95% CI |
| --- | --- | --- |
| Sex  Male  Female | reference  1.096 | 0.890-1.350 |
| Age (yrs)  <50  50-59  60-69  70-79  ≥80 | 0.616  0.700  reference  0.866  0.610 | 0.367-1.033  0.510-0.962*  0.672-1.117  0.430-0.865* |
| Histologic subtype  Adenocarcinoma  Non-microscopic verified | Reference  0.938 | 0.726-1.212 |
| Location of metastases  Liver  Peritoneum  Lung  Extra regional lymphnodes  Other  2 organs  3 or more organs | reference  0.756  1.497  0.847  1.460  1.301  1.678 | 0.490-1.165  0.905-2.477  0.490-1.464  0.716-2.978  1.007-1.680  1.163-2.422 |

Supplementary table 1c Multivariate binary logistic regression Patient characteristics in a twice high-volume hospital (n=507) vs once and three times high-volume hospital (n=1394)

*Abbreviations*

*95% CI= 95% confidence interval*
