# Supplementary material for: Volume matters in the systemic treatment of metastatic pancreatic cancer: a population-based study in the Netherlands
Source: J Cancer Res Clin Oncol. 2016 Mar 19;142(6):1353–60. doi: 10.1007/s00432-016-2140-5 (PMC4869755; doi:10.1007/s00432-016-2140-5)
Supplement: Supplementary file 4 — Supplementary material 4 (DOCX 49 kb) [file 432_2016_2140_MOESM4_ESM.docx]

| Variable | Odds ratio | 95% CI |
| --- | --- | --- |
| Sex  Male  Female | reference  0.721 | 0.512-1.015 |
| Age (yrs)  <50  50-59  60-69  70-79  ≥80 | 1.353  1.138  reference  0.790  0.474 | 0.704-2.600  0.731-1.770  0.517-1.206  0.223-1.007 |
| Histologic subtype  Adenocarcinoma  Non-microscopic verified | Reference  0.347 | 0.198-1.608* |
| Location of metastases  Liver  Peritoneum  Lung  Extra regional lymphnodes  Other  2 organs  3 or more organs | reference  0.898  1.317  1.839  0.316  1.035  0.739 | 0.472-1.711  0.575-3.018  0.958-3.531  0.042-2.367  0.682-1.572  0.408-1.540 |

Supplementary table 1d Multivariate binary logistic regression Patient characteristics in a three times a high-volume hospital (n=161) versus once and two times a high-volume hospital (n=1740)

*Abbreviations*

*95% CI= 95% confidence interval*
